# Deep learning analysis of sugar beet (*Beta vulgaris* ssp vulgaris) protease inhibitors interacting with identified trypsins from its primary pathogen, the sugar beet root maggot *Tetanops myopaeformis*

**DOI:** 10.1016/j.dib.2026.112748

**Published:** 2026-04-07

**Authors:** Sidra S. Rahim, Yerang Lee, Nadim W. Alkharouf, Chenggen Chu, Vincent P. Klink

**Affiliations:** a Department of Computer and Information Sciences, Towson University, Towson, MD 21252, USA; b USDA-ARS-NA- Northern Great Plains Research Laboratory, 1307N 18TH ST, Northern Crop Science Laboratory, Fargo, ND 58102, USA; c USDA-ARS-NEA-BARC, Molecular Plant Pathology Laboratory, Building 004, Room 122, BARC-West, 10300 Baltimore Ave., Beltsville, MD 20705, USA

**Keywords:** Plant pathogen, Insect, *Tetanops myopaeformis*, kunitz trypsin inhibitor, sugar beet root maggot, nematode, *Heterodera glycines*, stakeholder, trypsin, soybean, soybean cyst nematode

## Abstract

*Beta vulgaris* ssp. vulgaris (sugar beet) is one of two plants globally from which sugar is widely produced, accounting for 55% of U.S. sugar $1B (U.S.) and 35% of global raw sugar $4.6B), annually. Its top pathogen, *Tetanops myopaeformis*, is capable of causing total crop failure, making its study of utmost urgency. A *B. vulgaris* protease inhibitor, *Bv*STI (DV501688), one of 22 of its Kunitz trypsin inhibitors (KTIs) that target trypsin (Trp), is expressed during resistance to *T. myopaeformis* infection. The *T. myopaeformis* genome made BLASTp searches possible using the Trp protein XP_014094233 from the dipteran *Bactrocera oleae*, identifying 9 *T myopaeformis* Trps then used in Trp docking and cleavage studies. Trp docking analyses using the *T. myopaeformis* and Trp cleavage studies were done to determine the extent the *B. vulgaris* resistance architecture could be susceptible to Trp cleavage. KTI protein homologs then were identified in the model agricultural crop *Glycine* max (soybean) which undergoes infection by the root pathogen *Heterodera glycines* (soybean cyst nematode) that is used to better understand the *B. vulgaris*-*T. myopaeformis* pathosystem. The predicted interactions between *G.* max KTIs and *H. glycines* Trps are presented, including *Gm*KTI20, and *Gm*KTI30 that suppress parasitism by > 80%.

Specifications Table

**Table:** 

Subject	Omics
Specific subject area	Genomics
Data format	Raw, Analyzed, Filtered
Type of data	Table, Figures
Data collection	
Data source location	
Data accessibility	Repository name: NCBI; The Beta vulgaris Resource, Phytozome Direct URL to data: https://www.ncbi.nlm.nih.gov/bioproject/PRJNA1026092; https://bvseq.boku.ac.at/; https://phytozome-next.jgi.doe.gov/ The data has been deposited in Genbank SRA archive found at NCBI, or is available at the other sites, meeting the requirements for submission.

## 1 Value of the Data

• The *T. myopaeformis*, sugar beet root maggot (SBRM) genome, TmSBRM_v1.0, is providing data that researchers can use to understand its biology and aid stakeholders in its management. The data is used here to understand the resistance that its host, the *B. vulgaris* ssp. vulgaris (sugar beet), has to infection. This study began by identifying germplasm that exhibits natural resistance to *T. myopaeformis*. Through this identification, it was possible to begin understanding the process of resistance at the genetic and molecular levels. Furthermore, the identification of an actual *B. vulgaris* protein that has a defence function provided a polypeptide sequence for deep learning analyses to determine how it functions to inhibit pathogen effectors that have evolved to circumvent its host’s defence response.
• The SBRM genome annotation is allowing for future updated efforts to improve its sequence annotation and aid in other aspects of improving the understanding of insect biology, and agronomic impact(s). The information and methods will not only aid in future genome sequencing efforts, and annotations but will allow for comparative studies as is shown here to other crops (i.e. *Glycine* max [soybean]) and its crop pests (i.e. *Heterodera glycines* [soybean cyst nematode]) that significantly affect agriculture.
• The data that has been analyzed here has been deposited in public databases or is available as supplemental data and is available freely for use.
• The anticipated use of the data is scientific. The research presented here has either improved current annotations in two different crops, and two different crop pathogen species or provided the first annotation of the genes in a functionally transgenic manner. The annotations allow for gene function understanding and determining the breadth and scope of specific gene families (i.e. KTI and Trp). Importantly, the presented annotation permits target identification for their suppression or perturbation, or enhancement through overexpression, RNA interference (RNAi), mutagenesis, or clustered regularly interspaced short palindromic repeats (CRISPR)/CRISPR-associated protein 9 (Cas9) (CRISPR/Cas9)-mediated gene editing that synthetically modifies genes [[1], [2], [3], [4], [5], [6], [7], [8], [9], [10]]. The information also improves traditional breeding approaches. Consequently, the availability of the TmSBRM_v1.0 reference genome and annotation allow for a better scientific investigation of the insect. Furthermore, the comparative analyses with other important crop and pathogen genomes (i.e. *G. max* and *H. glycines*) provides a broad context to the results presented here that, otherwise, would not exist. The analysis provides 3-dimensional protein-protein interaction maps between the host, *B. vulgaris*, and its most significant pathogen, *T. myopaeformis*. The work has also provided the same type of data for the heterologous plant pathosystem *G. max* and *H. glycines* for its 35 KTIs. This latter work is important because *G.* max has an annual value of $31B, U.S., and $155B, globally, annually which is 31–34 times higher than sugar beet. Furthermore, *G. max* is the top U.S. export crop ($27B, annually), but global production is hampered by *H. glycines* which results in an annual loss of $1.5B, U.S., $23B, globally. Therefore, what is learned from the *B. vulgaris*-*T. myopaeformis* pathosystem has broad ramifications.

## 2 Background

*T. myopaeformis* (von Röder), the sugar beet root maggot (SBRM), is a devastating pathogen of sugar beet (SB), *B. vulgaris*, ssp vulgaris (*B. vulgaris*) [11,12]. SB is an important food crop, while also being one of only two plants globally from which sugar is widely produced, and accounting for 35% of global raw sugar with an annual farm value in the U.S. of $1B alone [11,12]. SBRM is the most devastating pathogen of sugar beet in North America with agricultural control of SBRM being limited by a scarcity of genetic knowledge [11]. However, recently a *de novo* sequenced and assembled draft genome and its annotation of *T. myopaeformis* was presented to aid in understanding and or generating resistance [13,14]. From the standpoint of developing resistance through transgenic and/or gene editing standpoint, *B. vulgaris* is challenging so experiments have been done in other significant crop plant systems to understand various developmental and/or plant disease concepts [15]. One of these pathosystems, the *Glycine* max-*Heterodera glycines* plant pathosystem is like the *B. vulgaris*- *T. myopaeformis* system in that the pathogen affects the root, making it a useful system for heterologous study [15].

## 3 Data Description

All conceptually translated *B. vulgaris, T. myopaeformis, G. max, H. glycines,* and *Bactrocera oleae* reference sequences are provided (Table 1; Supplemental data 1). A *B. vulgaris* protease inhibitor, *Bv*STI (DV501688), was identified in gene expression experiments to be induced by infection by *T. myopaeformis* during a resistant reaction [16]. Experiments presented here determined it to be a Kunitz trypsin inhibitor (*Bv*KTI) with subsequent BLASTp analyses identifying an additional 21 *Bv*KTIs. Since the role of KTIs is to inhibit pathogen trypsins (Trps) which facilitate infection, BLASTp searches of the *T. myopaeformis* genome, having 28,276 protein coding genes were done. The analysis was made possible using the Trp protein XP_014094233 from the dipteran *Bactrocera oleae* identified 9 Trps from the publicly available annotated genome of *T. myopaeformis*, BioSample accession: SAMN37733483, BioProject ID PRJNA1026092. The data are at the URL: https://www.ncbi.nlm.nih.gov/bioproject/PRJNA1026092. Deep learning analyses led to the identification of *Bv*KTIs that dock to the *Tm*Trps, revealing they do interact and could function in resistance.

**Table 1:** Protein sequences used in the analysis.

*B. vulgaris* KTI		*G. max* KTI	
Protein	Accession	Protein	Accession
*Bv*KTI1	Bv4_081010_nnrr.t1	*Gm*KTI1	Glyma.01G095000
*Bv*KTI2	Bv_06500_pdgi.t1	*Gm*KTI2	Glyma.01G096200
*Bv*KTI3	Bv_06520_zcfr.t1	*Gm*KTI3	Glyma.03G068200
*Bv*KTI4	Bv3u_069470_mnqs.t1	*Gm*KTI4	Glyma.06G219900
*Bv*KTI5	Bv_06530_ieoe.t1	*Gm*KTI5	Glyma.08G235300
*Bv*KTI6	Bv_06540_xxgs.t1	*Gm*KTI6	Glyma.08G235400
*Bv*KTI7	Bv_06490_cgoi.t1	*Gm*KTI7	Glyma.08G341000
*Bv*KTI8	Bv_06600_jufw.t1	*Gm*KTI8	Glyma.08G341100
*Bv*KTI9	Bv_06470_efpt.t1	*Gm*KTI9	Glyma.08G341300
*Bv*KTI10	Bv_06550_muoq.t1	*Gm*KTI10	Glyma.08G341400
*Bv*KTI11	Bv_06580_frjx.t1	*Gm*KTI11	Glyma.08G341500
*Bv*KTI12	Bv6_153,420_udjq.t1	*Gm*KTI12	Glyma.08G341600
*Bv*KTI13	Bv6_153,430_sfyg.t1	*Gm*KTI13	Glyma.08G341700
*Bv*KTI14	Bv6_153,400_criy.t1	*Gm*KTI14	Glyma.08G341800
*Bv*KTI15	Bv6_153,410_hihh.t1	*Gm*KTI15	Glyma.08G342000
*Bv*KTI16	Bv3_066450_dhqp.t1	*Gm*KTI16	Glyma.08G342100
*Bv*KTI17	Bv6_153,440_aich.t1	*Gm*KTI17	Glyma.08G342200
*Bv*KTI18	Bv6_153,590_kpiu.t1	*Gm*KTI18	Glyma.08G342300
*Bv*KTI19	Bv6_153,580_rnpy.t1	*Gm*KTI19	Glyma.09G092800
*Bv*KTI20	Bv8_192,190_tujs.t1	*Gm*KTI20	Glyma.09G155500
*Bv*KTI21	Bv_41,530_sisg.t1	*Gm*KTI21	Glyma.09G162500
*Bv*KTI22	Bv6_153,450_yxie.t1	*Gm*KTI22	Glyma.09G162700
		*Gm*KTI23	Glyma.09G162800
*T. myopaeformis* trypsin		*Gm*KTI24	Glyma.09G163000
Protein	Accession	*Gm*KTI25	Glyma.09G163700
*Tm*Trp1	g7808	*Gm*KTI26	Glyma.09G163900
*Tm*Trp2	g3594	*Gm*KTI27	Glyma.12G234700
*Tm*Trp3	g23695	*Gm*KTI28	Glyma.12G234800
*Tm*Trp4	g23693	*Gm*KTI29	Glyma.15G211500
*Tm*Trp5	g3592	*Gm*KTI30	Glyma.16G211700
*Tm*Trp6	g7809	*Gm*KTI31	Glyma.16G212100
*Tm*Trp7	g23699	*Gm*KTI32	Glyma.16G212200
*Tm*Trp8	g23186	*Gm*KTI33	Glyma.16G212400
*Tm*Trp9	g18287	*Gm*KTI34	Glyma.16G212500
		*Gm*KTI35	Glyma.18G191400
*Bactrocera oleae* trypsin			
Protein	Accession	*H. glycines* trypsin	
*Bo*Trp1	XP_014094233	Protein	Accession
		*Hg*Trp1	CAA74204
		*Hg*Trp2	CAA74205

**Fig. 1:**
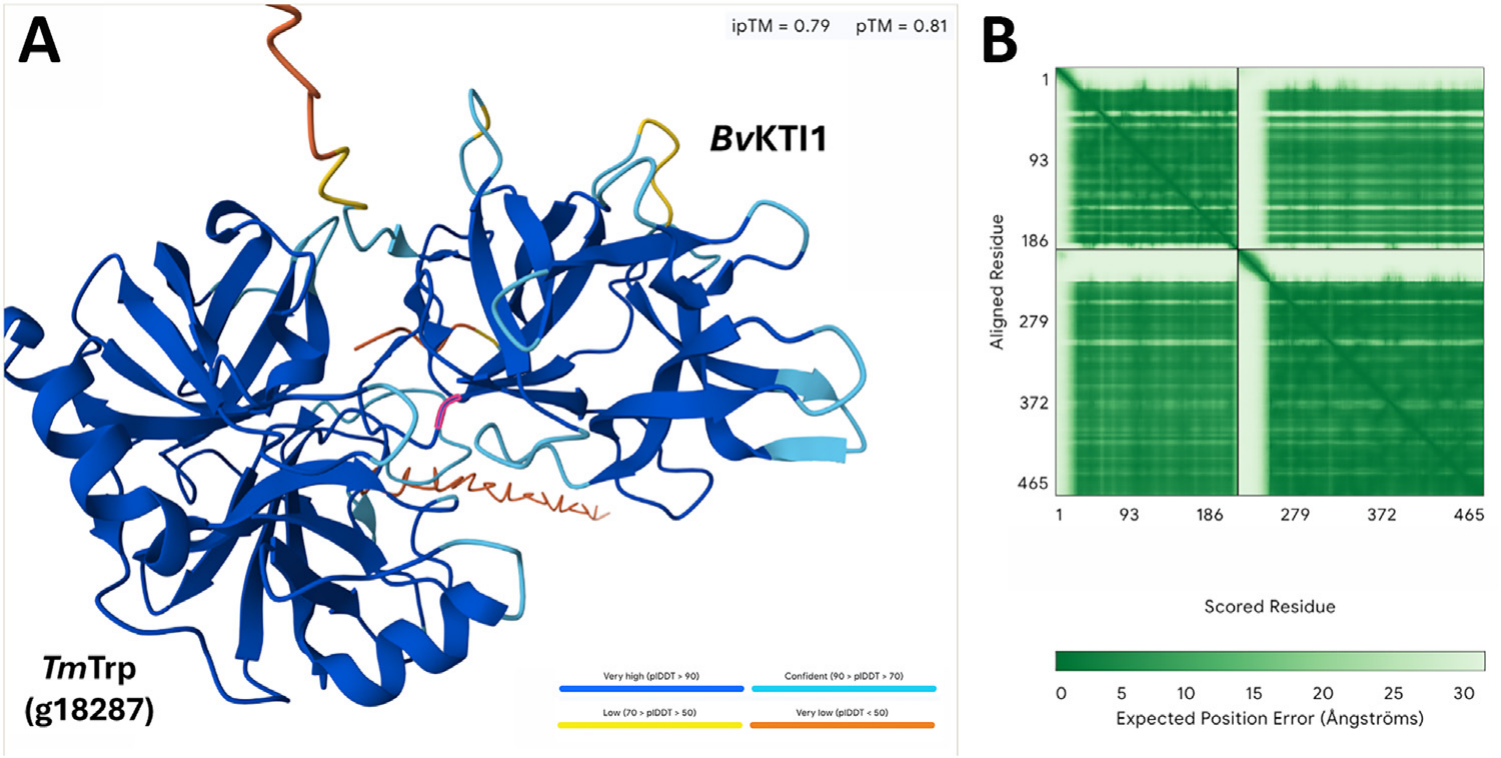
Predicted *Bv*KTI1 binding to *Tm*Trp g18287 (*Tm*Trp9). **A**. The image shows the interaction between *Bv*KTI1 and *Tm*Trp g18287. The highlighted amino acid, S87, marks the beginning of a loop located between β-strands 4 and 5 of KTI proteins. This loop corresponds to the binding interface and is critical for trypsin recognition of KTI. **B**. The predicted aligned error (PAE) indicates high confidence in both the residue positioning and the overall structural model.

The scope of the *Bv*KTI protein family as 22 *Bv*KTIs (*Bv*KTI1-*Bv*KTI22) in the RefBeet1.1 proteome is presented (Table 1; Supplemental data 1). This information may aid in understanding the scope of the *B. vulgaris* KTI gene family in two different iterations of its genome annotation (RefBeet1.1, and RefBeet3.0). The initial annotation to the RefBeet1.1 genome/proteome presented here was done due to gene expression analyses that used the RefBeet1.1 to examine infection by a root pathogen, the beet cyst nematode *H. schachtii*, a relative of *H. glycines* which parasitizes *G.* max [17]. The *Bv*KTI protein sequences then were used in BLASTp analyses of the updated RefBeet3.0 (Supplemental data 2). KTIs function to disarm Trps. The BLASTp analysis of the *T. myopaeformis* proteome with the dipteran *Bactrocera oleae* XP_014094233 Trp protein sequence identified 9 Trps (i.e. *Tm*Trp-g7808 [*Tm*Trp1], *Tm*Trp-g3594 [*Tm*Trp2], *Tm*Trp-g23695 [*Tm*Trp3], *Tm*Trp-g23693 [*Tm*Trp4], *Tm*Trp-g3592 [*Tm*Trp5], *Tm*Trp-g7809 [*Tm*Trp6], *Tm*Trp-g23699 [*Tm*Trp7], *Tm*Trp-g23186 [*Tm*Trp8], and *Tm*Trp-g18287 [*Tm*Trp9] (Table 1; Supplemental data 3). These 2 studies, for the first time, allow for deep learning analyses to determine whether the *Bv*KTIs and *Tm*Trps interact (Fig. 1; Supplemental data 4). This information is provided so the basis of the analysis is available.

**Fig. 2:**
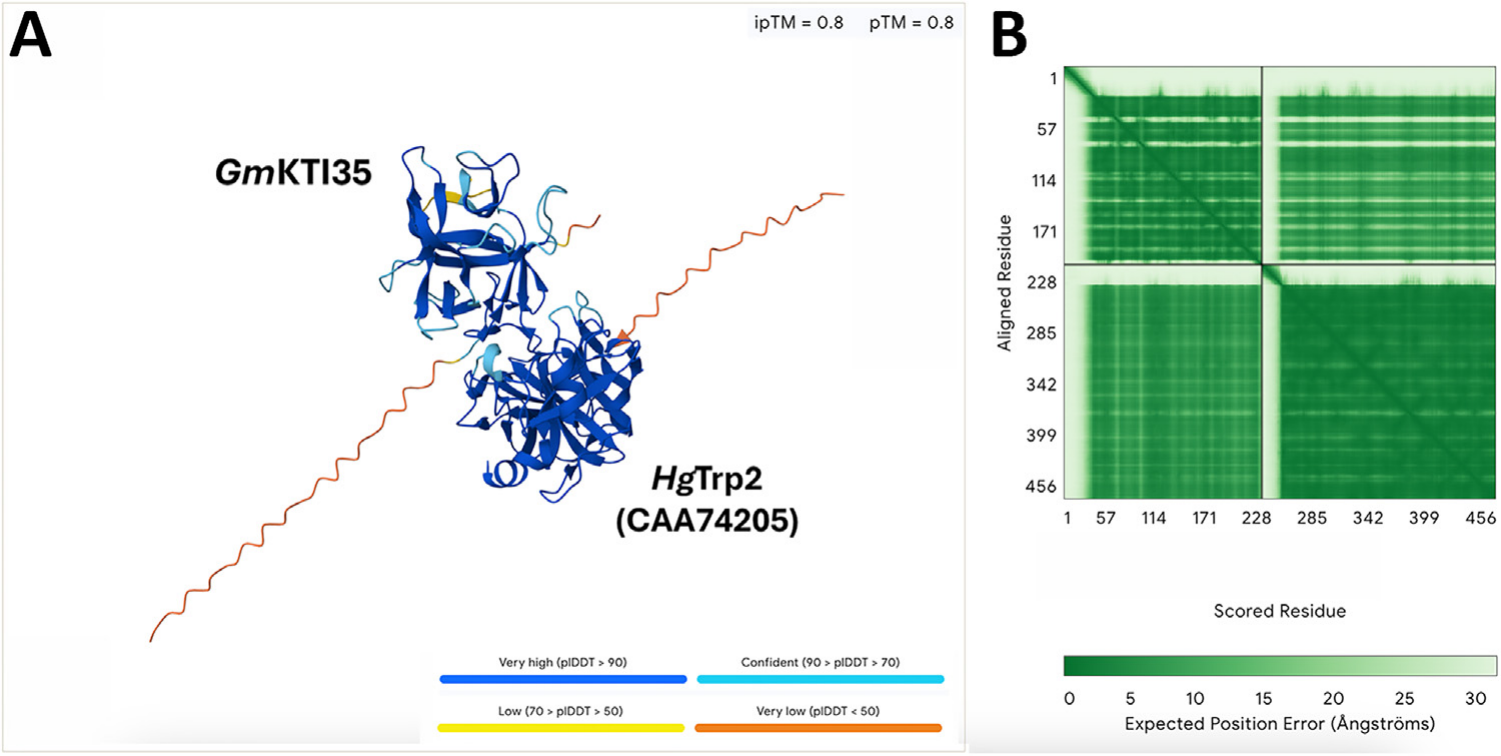
Predicted *Gm*KTI35 binding to *Hg*Trp (*Hg*Trp2). **A**. The image shows the interaction between *Gm*KTI and *Hg*Trp. The highlighted amino acid, S87, marks the beginning of a loop located between β-strands 4 and 5 of KTI proteins. This loop corresponds to the binding interface and is critical for trypsin recognition of KTI. **B**. PAE indicates high confidence in both the residue positioning and the overall structural model.

The analysis was repeated in the *G.* max proteome, using the *Bv*KTI1 protein sequence in BLASTp searches, identifying 35 *Gm*KTIs (*Gm*KTI1-*Gm*KTI35) (Table 1; Supplemental data 5). The *H. glycines* Trps have already been identified and are available in NCBI (Accessions: *Hg*Trp1 [CAA74204], and *Hg*Trp2 [CAA74205]) [18]. The identification of the *Gm*KTIs and *Hg*Trps allow for deep learning analyses to determine whether the *Gm*KTIs and *Hg*Trps interact (Table 1, Fig. 2; Supplemental data 6). This information is provided so the basis of the analysis is available. Performing BLASTp of each of the 9 *Tm*Trps in NCBI resulted in the identification of *Hg*Trp1 and *Hg*Trp2 (CAA74204), and *Hg*Trp2 (CAA74205), respectively (Supplemental data 7).

## 4 Experimental Design, Materials and Methods

The *T. myopaeformis* genome (https://www.ncbi.nlm.nih.gov/bioproject/PRJNA1026092) was *de novo* sequenced and archived at Genbank ([[14], [19]]). The genome sequence was obtained from NCBI [14]. Alkharouf et al. [14] used the pipeline Flye, version 2.9.2, to assemble the PacBio HiFi DNA reads using default values, except for setting the –asm-coverage argument to 50, to reduce memory consumption. The gene finding tool AUGUSTUS 3.5.0 [20] was used for gene prediction analysis in these contigs with the complete gene option enabled and default set for the rest of the parameters. *Drosophila melanogaster* is the most closely related genetic model that was most closely related, phylogenetically, to *T. myopaeformis* so it was used as the reference species to *T. myopaeformis* TmSBRM_v1.0. The identified genes were used to perform a blast against the non-reductant (NR) database to predict genes. For generating the gene functional annotation, the predicted genes were functionally annotated using Blast2GO 6.0 using default values [21]. The gene model was blasted as blastp against the NCBI NR protein database. InterproScan 5.67–99.0 [22] was used under default values for domain finding. Then GO mapping and annotation was performed under default values using GeneOntology 2024–03–28 [23]. BLASTp of the *T. myopaeformis* proteome used the *Bactrocera oleae* Trp XP_014094233 in default settings to identify its Trps. BLASTp queries were done in the *B. vulgaris* RefBeet1.1 and RefBeet3.0 proteomes at https://bvseq.boku.ac.at/ set on default using DV501688 (Bv4_081010_nnrr.t1) *Bv*STI (*Bv*KTI1). The *G.* max proteome BLASTp was done in Phytozome at https://phytozome.jgi.doe.gov. BLASTp of the *G.* max proteome was done with the *B. vulgaris Bv*KTI1. The foundational *H. glycines* Trps were extracted from NCBI, https://www.ncbi.nlm.nih.gov/ (Accessions: CAA74204, and CAA74205).

The 3-D protein-protein interaction studies using deep learning employed AlphaFold, a widely used tool for protein-protein docking on default settings ( [24]. Negative control protein-protein binding assay employed the *Aequorea victoria* GFP (accession: P42212.1). Trp cleavage prediction was performed by using PeptideCutter [25]. PeptideCutter was set at a lowest cleavage displayed option of 100% [25].

## Limitations

The KTI-Trp protein-protein interactions are bioinformatic in nature and are not presenting actual in vivo interactions leading to the disarming of the pathogen Trp.

## Ethics Statement

The authors have read and follow the ethical requirements for publication in Data in Brief and confirming that the current work does not involve human subjects, animal experiments, or any data collected from social media platforms.

## CRediT Author Statement

**SR** Methodology; Software; Validation; Formal analysis; Investigation; Resources; Data Curation; Writing - Original Draft. **YL** Methodology; Software; Validation; Formal analysis; Investigation; Resources; Data Curation; Writing - Original Draft. **NA** Methodology; Software; Validation; Formal analysis; Investigation; Resources; Data Curation; Writing - Original Draft. **CC** Investigation; Resources. **VK** Conceptualization; Methodology; Resources; Visualization; Supervision; Project administration; Funding acquisition; Writing - Original Draft

## Data Availability

NCBITetanops myopaeformis genome (Reference data).

## Appendix Supplementary materials

Supplemental data 1. Protein sequences used in the analysis. Image, application 1

Supplemental data 2. BLASTp results of the *B. vulgaris* RefBeet1.1 proteome with DV501688 Bv4_081010_nnrr.t1 (*Bv*KTI1) identifies the *Bv*KTI protein family in BLASTp searches of the RefBeet1.1 proteome using the conceptually translated DV501688 under default settings. Image, application 2

Supplemental data 3. BLASTp results of the *T. myopaeformis Tm*SBRM_v1.0 proteome with the dipteran *Bactrocera oleae* XP_014094233 Trp identifies the *Tm*Trp protein family. Image, application 3

Supplemental data 4. Deep learning analyses to determine whether the *Bv*KTIs and *Tm*Trps interact. The ipTM (interface predicted template modeling), derived from the predicted template modeling (pTM) score, which evaluates the overall accuracy of the predicted structure, measures interface quality between two proteins, showing how well the predicted structure aligns with the actual structure, obtained from the predicted protein-protein binding analysis done in AlphaFold. An ipTM > 0.7 is considered a strong likelihood of an interaction; ipTM 0.5–0.7 is considered a possible likelihood of an interaction; possible; ipTM < 0.5 is considered a weak/unlikely possible likelihood of an interaction. Image, application 4

Supplemental data 5. BLASTp results of Bv4_081010_nnrr.t1 (*Bv*KTI1) protein sequence in the *G.* max proteome identifying *Gm*KTIs. Image, application 5

Supplemental data 6. Deep learning analyses to determine whether the *Gm*KTIs and *Hg*Trps interact. The ipTM measures interface quality between two proteins, showing how well the predicted structure aligns with the actual structure, obtained from the predicted protein-protein binding analysis done in AlphaFold. An ipTM > 0.7 is considered a strong likelihood of an interaction; ipTM 0.5–0.7 is considered a possible likelihood of an interaction; possible; ipTM < 0.5 is considered a weak/unlikely possible likelihood of an interaction. Image, application 6

Supplemental data 7. BLASTp of each of the 9 *Tm*Trps in NCBI resulted in the identification of *Hg*Trp1 (CAA74204), and *Hg*Trp2 (CAA74205), respectively, confirming their relatedness at the primary amino acid sequence level. Image, application 7
