# Supplementary material for: Deep learning analysis of sugar beet (Beta vulgaris ssp vulgaris) protease inhibitors interacting with identified trypsins from its primary pathogen, the sugar beet root maggot Tetanops myopaeformis
Source: Data Brief. 2026 Apr 7;66:112748. doi: 10.1016/j.dib.2026.112748 (PMC13099927; doi:10.1016/j.dib.2026.112748)
Supplement: Supplementary file 1 — Supplemental data 1. Protein sequences used in the analysis. [file mmc1.docx]

***Beta vulgaris* KTIs**

BvKTI1

>Bv4_081010_nnrr.t1 length=198 cDNAcoverage=100%

MASIFLKSTTTALLLIFSTLCIATAVVIQDTDGDALRNGGQYYIIPVSAGFQGGLTLKSKADNSPCPLYITRDKVETSRGIPVTIASPYRIAIITSSIPIGIVFTNTPNVCMQPLGWQVVADEKTGQSYVATGGNGFGFNPTESFDIQQIEGNNNVYKIRFAGESEVGFFEKDGLLGITNEIPLPVVFQKAFDVLAMV

BvKTI2

>Bv_06500_pdgi.t1 length=198 cDNAcoverage=100%

MASIFLKSTTTLILIFSALCIATAVILDTDGDALRNGGQYYIIPRSLGFGGGLTLNSKAGNTPCPLYITRENDETSPGIAVTIASPARIGIITSSLPISIVFNDIPNICMQPLWWQVIPDGTTGQSYVATGGSGIPFNPTETFSIEPIEGNIYKIRNGVGSDQARDVGFFENDGLLGITNDIPLPVVFRKAFDVLAMV

BvKTI3

>Bv_06520_zcfr.t1 length=199 cDNAcoverage=100%

MASIFLKSTTTVLLLISALSIATAFILDIDGDPLYNGGQYYIIPQSLGFGGGLTLKSKADSLPCPFYIGRENDETSHGMPVTIASPFKIGIITPSLPISIVFKGDGNPNICMQPLGWQVIADATTGQSYIATGGSGGAFNPTTTFSIEQIGININMYKIRNAGGSDVGFFEKDGLLGLTDDIPLHVVFRKAFDVLSMKV

BvKTI4

>Bv3u_069470_mnqs.t1 length=205 cDNAcoverage=100%

MASIFLKQTISLISLTLLLIFSALSISTAFVLDTDGEPLVNGGQYYIIPQSAGIMGRGLTLISKPDASPCPLHITRDKDETSLGIPATITSPFKVEFITDSVPINIVFKDTPNICVQSLAWQMIPDETTGQFYVATGGSGFLPTESFMVVKIGDKNVYKMRYIGGFDEKLYIGIFEKDGLLGSTNEIPLPVVFKKTSFDVLRMTN

BvKTI5

>Bv_06530_ieoe.t1 length=162 cDNAcoverage=100%

MQYYIIPQSLGFGGGLTLKSKAGTSPCPLYIGRENDETSPGIPVRISSPFMIATITPSLPINLVFKDTPSICMQPLGWQVIPDGTTGQSYVATGGSSIPFNPTETFGIEQIGDNNVYKISNRGTFGEARYVGFFEKDGLLGITNDIPLPVVFRKAFDVLSKV

BvKTI6

>Bv_06540_xxgs.t1 length=74 cDNAcoverage=63.6%

MNPEIRWNQIECPCQPLGTIDCGYYVCRYMLETVQLRRLLISTIKALVQTILQRKLMNSRSCGLHMSEINMKHK

BvKTI7

>Bv_06490_cgoi.t1 length=204 cDNAcoverage=100%

MGSIFLKSTTTLLLIFSTLSIATAFILDTDGEPLLNSRKYYILPQSLGFGGGLTRTTKNTDLPCPYYITRDNDETSNGMPLIISSPFRILYTPLSSPVYIAFEEMITVCIQSMGWRLIPDDSTGRSYVGTGASGFDLTQRFTIEHAGSEFENNVYKIRYIGESGEGRDVGFFKEDGLLGITDDIPLTVMFKKAFNVLEETTSKM

BvKTI8

>Bv_06600_jufw.t1 length=209 cDNAcoverage=100%

MASIFLKSTITLLLIFSTLSVAIAVVLDTDGEPLFNQVNNSRYYILPQTIGIGGGLTRTTKNPELPCPYYITRDNDETSSGMPLYISSPLKILFIPLSSPVRISFEEMPTICIQESMGWRVISDDSTGRLYVSTGAGRGPGRFTIEQAESESSNNVYKIRYIGALDSEAGAGDLGFFKEDGLLGITDDIPLTVVFKKAFDVPEETTTSM

BvKTI9

>Bv_06470_efpt.t1 length=201 cDNAcoverage=80%

MGSNFLKSITTPLIFSTLSIATAFILDTDGEPLFNSRKYYILPQSLGFGGGLIRTTKTTDLPCPYYITVDNDDTSYGMPLIISSPFRILYTPLSSPVYIAFEEMITVCIESMGWRLNPDNSTGRSTGASGFDLTQRFTIEHAGSEFENNVYKIKYIGASGEGRDVGFFDFNEDGLLGITDDIPLTVMFKKAIILEETTSRM

BvKTI10

>Bv_06550_muoq.t1 length=86 cDNAcoverage=100%

MQSLGWQVIADATTGQSYIATGGSGGAFNPTSTFSIEQIGVNMYKIRNAGGSDLGFFEKDGLLGLTDDIPLHVVFRKAFDVLSMKV

BvKTI11

>Bv_06580_frjx.t1 length=134 cDNAcoverage=60%

MGPTLPQSLGFGGGLTTTTKNTDLPCPYYVTRDKDETSNGMPLIISSPFRMLQIPLSSHVYIAFEQMITVCIQSMGWRLIPDDSTGRSYIGDSGEGRDVGFFKEDGLLGITDDIPLTVMFKKALNVLEETTSGM

BvKTI12

>Bv6_153420_udjq.t1 length=206 cDNAcoverage=100%

MTHVIISAATIFLFLYLSPLTSTADNTAVLDINGRPLQAGYNYYILPVIRGRGGGLTMASKNATELCPLYVAQEDHEVSNGLPLKFYPVNPKDKRISLSSDLNFVFDAATTCVQSTGWSLTIEMETGRRYVGTGGEIGNPGVETVDNWFKIEKDGSGKYDYKIVYCPGVCNFCKVMCGDVGVFIEKDGRRLLGFSDQPLLVMFKKA

BvKTI13

>Bv6_153430_sfyg.t1 length=208 cDNAcoverage=100%

MSHVVILSAATIFLFLCLSPLTSTAANIAVLDINGRPLQARSNYYILPVIRGRGGGLRMTPKNATQLCPLAYVAQEGSELANGLPLKFYPVNPKDKTISLSTDLNFVFDAATICVQSTQWRLAFDEVTGRRYVGFGGEIGNPGGNTVSNWFKIEKAETGKYDYKIVFCPGVCNFCKVACGEIGVFVEKDGRRLLGINNQPLLVMFKKA

BvKTI14

>Bv6_153400_criy.t1 length=262 cDNAcoverage=20%

MTQDIIISATTFLLLFLSPSWGNAADTSVLDTEGRPLRAKSRYYILPASQGQGGGITVSQKNQTTLCPLYVSQESQEIYLGLSVWFLPSKHNQRLIYISSDINILFNMVNICLQSAAWKLSIDHTTWRKYVATGGAIGNPGEETVSSWFKIEKVKSGSYEYDYNYKIMYCPNVCSFCMVMCGDVGVFVQDDGTSSIKDIQDLVLLRLGWWIKGWYEEFPYSAIDIQRTPSCLLWNGFAAVHPPIKSLSTPVIWNPPVVNHLK

BvKTI15

>Bv6_153410_hihh.t1 length=211 cDNAcoverage=100%

MTPHSILSILTILLFLLIAPLSTTTAAAKTTTVLDINGRPLKTDSTYYILPVSRGRGGGLAMAPKNATESCPLYVAQENHEVSNGLPLKFFPTNPNDHDKIPLGYDVNIVFDAATICVQPTGWMLAFDEASGTRYVGIGGTIGNPGVDTLSNWFAIEKAGSGLYDYKIRFCPAVCIFCTVMCGDVGVFIGEGGTRFLALTDRPLLVRFKKA

BvKTI16

>Bv3_066450_dhqp.t1 length=212 cDNAcoverage=66.7%

MATHFISSTLVLATFLLFVSPPAAVAQVINIFDMDAEPVQAGKLYYILPVVQRQGGGISTAPKNANESSCPLYVVQEKDTTSLGLAVTFNLALPNTTNVTFSADMNIVFGQAINCVESPVWTLALDEPTGRRYVALGGLGMARGPKAVNNWFRIERFFPRFHFDYKFVFCPTRVICPTCQNSCGDLGVFVRDDGTWVLGVGAPPLRIKFKKA

BvKTI17

>Bv6_153440_aich.t1 length=221 cDNAcoverage=100%

MSHLILQLSVTILLIFSLPTNIATKTTDDSLVLDVDGNPIEVGSAYYVRTELSKAGIGGGLVTASKPNHTQCPQYVAQLAYGFEGEIPVTFYPSSSSQKFIHISSDINIIFNTTSNVCSQGAWQLTPDASNGNLYLSTGGVIGNPGSLTTANWFKIAKSPYDPNFYQLEYCPDTKTVASPTGDIVCGAIDALDSTDDFLLMWLGLKTIRPDFFHWLSFVKA

BvKTI18

>Bv6_153590_kpiu.t1 length=228 cDNAcoverage=100%

MSHLHFIFSFFLLSLLLSPSITSAAESLLDTDGNPLSSSGSYYVLPVSWGAGGGLNIAAIQDHHTRCPYYSVVQSQDDRCGSNLGLPVTFTPSDLEEGQNITLATDFSIDFNIRPPHAPRFCDQPTTTWEMVSVDGPNNIGAQLGSFHHGGLTRGGGDDKQGSLFKMVKNKAYGYRLRYCPTNSSKNSPLKNVVCGDLAPVYDRRLGVRVLSLVDDSYRPFEIMFKKA

BvKTI19

>Bv6_153580_rnpy.t1 length=221 cDNAcoverage=100%

MISSAQLIYDTSGDIVTSSNAYYLMPVSRGSGGGFNIAAVQDYHTRCPYYSVVQSPHDRCGSYFGLPVKFSRSSTSTTADSNHQEVNVTLSTDFTINFSTYTPHAPRYCDQPTTMWNMVSRGGSGDDMSVELGMVHFGGLNHDDDDDDNDDDDDRQSSLFKIVKNKAYGYRLRFCPSNSNSSALKDVVCGDLAPVYDRRLGVRVLRLVDSKSPAMIMFHKA

BvKTI20

>Bv8_192190_tujs.t1 length=221 cDNAcoverage=100%

MKHLILFLASLYCAVTAAAITTITADDNSVYTVIGDPLLAGQDYYVVPYNYGDVGGGLTWKLKQELLAICPHYYVLQVEDNLNFGEPTAFFPSNSRQNQITLSNKLNILFSKVSPPASPPFVCPASSNVWKVVADDSTGIVYVELGGRKGRSRDTNSWFTIEELDEGGYQIKYCDSTKRKTASVCGGLGIVSLNGGRYLGVNATNPVAFAFETQQIFVKTA

BvKTI21

>Bv_41530_sisg.t1 length=235 cDNAcoverage=100%

MAHQFILQAIATILVTLLFLSCPITAGSPVLDLDGNPLEVGSQYYVLTAGWGAAGRGGLTDTFKPPSVCPSYVAQHRLWTNNGRPVTFYPSDSSQKQIIQGDEMNIAFGIIPLCRSTGIWRLTFDNETQVPYVATNGVIGNPGTFETFGNWFKIEKAFGDSSYKIVFCFVEPVPAPGNTNLVGRRRHCEQLDATQKGPNGDLSYLSLVPLDQNLPFFGYVFKKVDTSSATTTAFY

BvKTI22

>Bv6_153450_yxie.t1 length=239 cDNAcoverage=100%

MKHLFLPLATTTTLLLFLLLLLSTPPTNATDITVYDAIGEPLVAGQSYYLVPYEYIDVGGGGLTWKQKVKSEGNCAPYYVSQQNENTDFGSQITFFPQNTRQTEITTSTELNIVFDSYSPPYNCPGSSNVWRTALDRATQTHFIELAGRKGTRDVRTWFTIQELGPDLGGYKIRYCPRHRQADCGDLGIFPRANVIDTRWLCVNGSNPLAFMFESEELFLKNKARRSVPTYNLGTTYAE

***G. max* KTIs**

>Glyma.01G095000 (GmKTI1)

MKSTIFFALFLVCAFTISYLPSATAQFVLDTDDDPLQNGGTYYMLPVMRGKGGGIEVDSTGKEICPLTVVQSPNELDKGIGLVFTSPLHALFIAEGYPLSIKFGSFAVITLCAGMPTEWAIVEREGLQAVKLAARDTVDGWFNIERVSREYNDYKLVFCPQQAEDNKCEDIGIQIDDDGIRRLVLSKNKPLVVQFQKFRSSTA

>Glyma.01G096200 (GmKTI2)

MKSSTLFALFLLSAIFTSYLPSATADVVLDTDGGVLQNGGQYSVLPVMRGSGGGLVVRATGNERCPLTVAQTRNELDKGIGTIISSPLRVAVIAEGHPLSISFGFFPVMPSCIPLTGDWGIVDGLPEGPAVKLAEYKNIVDGWFKIEKAHPLGYKLLFCPLLEGSTCGDIGIQTDDDGIRRLVVTKNNPLLVQFQKIGWILTKNNLLLPGSE

>Glyma.03G068200 (GmKTI3)

MKTINSLFGLLFLLLIAFTMKPAVASGVHDTDDRMLENGGDYFILPSFDEEGGGVTLASIGVTHPLAVVQSSSKNHLGLPASIKTKETMIIFTDEMVSIKFTNVPDYNNASTWTLVMDEFVKVWYVGIGNVADYPSHRIKTGNFQIKEDNFDYKLVFCADANTTSCEDVGVYVDGEQNRRLVLSEVGGVAVKFMNADKSRVAFD

>Glyma.06G219900 (GmKTI4)

MKSTLFALFLLCALTSYLPSATAGDVVDTDGNPVENGGTYFVLPTIILNGGGIEYATFGNETCPVTVAQSRDQFCKGFPITISSPARIRHISEGLSLNIGFTFASPCSPASEWTIVKDQPEGLAVKLTGFKNTVPGVFTLKRVPADEIIGYNILFCPLDNNPCGYVAVHFDQFRNRRLVVSEVQEDGLWVMFQKLSYASGLTLSSA

>Glyma.08G235300 (GmKTI5)

MSMKLLLASLSISVWLFMATLSLAQSNNRYVFDTHGDPLETDDEYYIRPAITDNGGRFTLINRNRSCPLYVGLENTDTPQGYPMKFTPFANKDDDDNLRVNTDLKVTLVQVSTTCVQSTEWKLGENDTRSGRRLIVTGRDNGIQSAGNYFRIVETESVGIYNIRWCPTEACPTCRFICGTGGILRENGRILFALDGTTLPVVFQKKDD

>Glyma.08G235400 (GmKTI6)

MSMKLYASLTLTVWLFMATFSRAQYVIDTNGEPVDNDDEYYIRPAITDNGGRFTLINRNGSCPLYVGLENTDTPLGYPVKFTHFALNVQDEDIRVNSDLRIEFVEVSTTCVQSTEWRVGENDTRSGRRLIITGLDDNFGSIGNYFRIVETQSVGIYNIEWCPMEICSDCGFVCSTGGILREDGRIFFALDGTPLPVVFQKKDD

>Glyma.08G341000 (GmKTI7)

MKSTSLFAIFLLCAFTSYLPSATAQDVLDVDGDPIRNGFIYYVLPAIRGNGGGIERAALGKDTCPITVVQSPNPNSKGLEIKFESAYPAYYINETLILQIKFSYPQQCERKNPWWAISKDISEGPPAIKLSGFHGTELGWFKIQKASKSCDSNDYKLVFCQYDETWCLDVGIYVDRQGNRRLVLAVTGEPFLVHFHKISSSTA

>Glyma.08G341100 (GmKTI8)

MKNTALFTIFLLLAFTSYLPSATAASVYDTDGNILQKGRTYFVLAATRGNGGGIEFAATGNDTCPLTVVQSPSGDSIGFPIRFSYPFIETLDDILEGFGLEIRFAYRPPCSPSSIATTKWTIVNDAVKLTKTSSSIIPGFFYIWGASPNSKDYKLEFCDLDNIKCGDVGIHTDDDGSRRLVITQNDPLLVHFQNTRSSSA

>Glyma.08G341300 (GmKTI9)

MKNTIFFALFLVCAFTSYLPSATADTIFDINGDFVRNGGTYYILPVIRGDGGGIEFAATGNETCPLTVVQSPLEVSKGLPLIISSPFEILSIQEGLILNIGFTFVPPCALIPSEWTTVKGLPEGLAVKLTGYENKVPGWFKIERVSLEFNDYKLVFCATEDSTCVDIGVYIDGEGNRRLVVTENNDPLLVHFKKVEVESSATA

>Glyma.08G341400 (GmKTI10)

MKSTTSLALFLLCALTSSYQPSATADIVFDTEGNPIRNGGTYYVLPVIRGKGGGIEFAKTETETCPLTVVQSPFEVSKGLPLIISSPFKILDITEGLILSLSFTYVPPCASTPSRWTVILKGLPEELHVKLTGYKNTIDGWFRIQRASSESNYYKLVFCTSNDDSSCGDIVAPIDREGNRPLIVTHDQNHPLLVQFQKVEAYESSTA

>Glyma.08G341500 (GmKTI11)

MKSTIFFALFLFCAFTTSYLPSAIADFVLDNEGNPLENGGTYYILSDITAFGGIRAAPTGNERCPLTVVQSRNELDKGIGTIISSPYRIRFIAEGHPLSLKFDSFAVIMLCVGIPTEWSVVEDLPEGPAVKIGENKDAMDGWFRLERVSDDEFNNYKLVFCPQQAEDDKCGDIGISIDHDDGTRRLVVSKNKPLVVQFQKLDKESLAKKNHGLSRSE

>Glyma.08G341600 (GmKTI12)

MKNTTSFALFLLCAFFTSYLPSATGAFVLDSDGNPLENGGTYYVPAPNNCGGIEYTTSGNETCPITVVQHHDPTCKGFPITISSPARIRYISEGLNVNIGFTFRPPPCAPSSLWTVLKDQSEHPDPYEGVILPVKLNNEDNSNNTVPGWFKIQKLPLDFITTYYIVFCPLDQSPCWSVGSNFDQYGNRRLVVAQFARRQDIEFQKLSYASRSTHSSA

>Glyma.08G341700 (GmKTI13)

MKSTTLFALFLLCALTSSYLPSATADVVLDMDGNPLGNGWQNEYFMLPVTRGSGGGIALAATGNERCPVTVVQSHIERDKGYAATFRAPFHLPFIAEGLPLTISFDNFEVLPRCVPTPLWWAIVDGLAEGPAVKIEYRDIVEGWFKIQKAYPLGYKLLFCPVPLEDSTCGDIGIYTDDKGFRRLVVTKNKPLVVQFQKFDPLAKNNLVLPASE

>Glyma.08G341800 (GmKTI14)

MKNTIFSALFLLCAFTTSYLPSTTAVVDTNGDILQNPGTYFILSVFRPGGGVEFAATGNETCPLTVVQTLFGRGFPVILSSQLRIPIIGEGQLFSILFRIVPWCATTPSKWTIVEGLPESPAVKLTGYDNTVPGEFKIEKANPFHNDYTLLFCPAGEESKCGHIGIHFDDDGNRRLVVSEENILRVQFQKFGSSAPDEASLALKKHHVLSVSE

>Glyma.08G342000 (GmKTI15)

MKSPTIFILFLLSAFYTSLLPSATAQVVVDMEGNDLQNGGKYYVLPVIESSYGGIRVAATGKERCPLTVVQSADPYDKGIATIISTPYRVPVIREGFPLNITFGNFAVILPCVPLRSEWTVVNGQPEGPAVKIGSPPNAENGWFEIEKLLTSGYKLVFCTRPERSYCQDIGIHVDDENHARLVLTNDDPLVVEFLKYDPFWPQNNLVLPTSQ

>Glyma.08G342100 (GmKTI16)

MKNTIFSALFLLCAFTTSYLPSTTAVVDTDGDILQNPGTYFILSVFRPGGGVEFAATGNETCPLTVVQTLFGRGFPAILSSRLRIPFIGEGQLFSILFRIVPWCATTPSKWTIVEGLPESPAVKLTGYDNTVPGEFKIEKANPFHNDYTLLFCPAGEESKCGHIGIHFDDDGNRRLVVSEENILRVQFQKFGSSAPDEASLALKKHHVLSVSE

>Glyma.08G342200 (GmKTI17)

MFRESLFALFLLSAFSSSLLPLTTAQDYVLDTDGHAVENHGTYYLLPAKSGSGGGGIEVAATGKESCALTVVQSLNEDSMGLPLKLSSPSITTSHFTEYTSLSIEFTSAPAPCSSASEWTVVKGLPEGRAVKLNDYGNTVEGDFAFVCAKREFYRCNKSYQLIFCPYGLMRCEDVGISIDDDGNRRLVISDGNPFLFKLQKVGSSSSA

>Glyma.08G342300 (GmKTI18)

MKRTPILFPLFFLLCAFTSYLPSATADDDHVYDTDGDKLQYGVNYFVLPVIRGNGGGIQVAKAGNETCPLTVVQSGNELSEGLPIKIASRSAGVAFITQGQLFKSIQFGVFPSTLRPGCPPSPIPSKWDPPSKWTIVEGLPERGLAVKLVGYQNRVSGWFSIVKVADDASSSSVGYKLVFCLWPEEEVMIHLCKNVGIRTDGKGIRRLVLSENTPLVVQFQKFRSALALNNHVLSASE

>Glyma.09G092800 (GmKTI19)

MKKVSALAFSILFLAFTIEPFIGIAEAAPEVVLDTSSHKLRIGVKYYILSVFKGKGGGLTISSSDNNTCSFFVRSLKSQRHPVTFTPYNAKSGVILTSIDLNIKSYP

>Glyma.09G155500 (GmKTI20)

MKPTLLLSLSFLPLFAFLALSEDVEQVVDISGNPIFPGGTYYIMPSTWGAAGGGLKLGRTGNSNCPVTVLQDYSEIFRGTPVKFSIPGISPGIIFTGTPLEIEFAEKPYCAESSKWVAFVDNEIQKACVGIGGPEGHPGQQTFSGTFSIQKYKFGYKLVFCITGSGTCLDIGRFDAKNGEGGRRLNLTEHEAFDIVFIEASKVDGIIKSVV

>Glyma.09G162500 (GmKTI21)

MKMTLVTLVLIVALSTKALLGAAGPAPEQVLDTSGKIVRARSSYYIVPASPDLGGLDMASTGADCPLDVVAVDGYQGQPLIFTPVNFNKGVIRVSTDLNIYFPVGTSCPQTTAWKLKDYDYSTSQWFVTTGGDFGNPGSQTVANWFKIEKYEDAYKLVYCPSVCNDCSYPCSDIGIYQDEYGKRLALSSEPYKVKFQRA

>Glyma.09G162700 (GmKTI22)

MKMTLVTLVLIVALSTKALLGAAGPAPEQVLDTSGKIVRARSSYYIVPASPDLGGLDMATTGADCPLDVVVVDGYQGQPLIFTPVNFNKGVIRVSTDLNIYFPVATSCPQTTVWKLKDYDYSTSQRFVTTGGDFGNPGSQTVANWFKIEKYEDAYKLVYCPSVCNDCSYPCGDIGIYQDEYGKRLALSSEPYKVKFQRA

>Glyma.09G162800 (GmKTI23)

MKMTLVTLVLIVALSTKALLGAAGPAPEQVLDTSGKIVRARSSYYIVPASPDLGGLDMASTGADCPLDVVAIDGYQGQPLIFTPVNFNKGVIRVSTDLNIYFPVATSCPQTTVWKLKDYDYSTSQWFVTTGGDFGNPGSQTMANWFKIEKYEDAYKLVYCPSVCNDCSYPCSDIGIYQDQYGKRLALSSEPYKVKFLRA

>Glyma.09G163000 (GmKTI24)

MKMTLVILVLIVALSTKALLGAAGPAPEQVLDTAGKIVRARSSYYIVPASPDLGGLDMASTGADCPLDVVAVDGYQGQPLIFTPVNFNKGVIRVSTDLNIYFPVGTSCPQTTVWKLKDYDYSTSQWFVTTGGDFGNPGSQTVANWFKIEKYEDAYKLVYCPSVCNDCSYPCSDIGIYQDEYGKRLALSSEPYKVKFQRA

>Glyma.09G163700 (GmKTI25)

MKTKLLAFLLFFALTTKPLLLGAAGAAPEPVIDTSGKKLRADANYHIIPAVPFTICGFVSCFTGGGLSLDSIDESCPLDVIIEKANEGLPLRFSPVNTKKGVIRVSTDLNIFFSDSDERCPHHSTVWMLDQFDASIGQTYVTTGGVVGNPGEHTILNWFKIQKYEDAYKLVYCPRVCPSCHHLCKDIGMFVDANRRMHLALSDDPFKIKFKEA

>Glyma.09G163900 (GmKTI26)

MKVSPLAFSILFLSFTIELFIGIASAAQEPVLDTSGQKLRTGVKYYILPVFRGRGGGLTVSSSGNNTCPLFVVQEKLEVSKGTPVTFTPYNAESGVILTSTDLNIKSYVKSTTCDKPPVWKLLKVLTGVWFLSTGGVEGNPGVNTVVNWFKIEKAEKDYVLSFCPSFAQTLCRELGLYVGDDGNKHLSLSDKVPSFRVIFKRA

>Glyma.12G234700 (GmKTI27)

MSTKLIETSLSLMVWLVIATSAIAQSDNPPVLDTEGHPLEPGRDYYITPAVTDIGGRATIVDNGTCPLFVGQENTFVEESFAVFFTPFAKEDDVVKVNRDFQVAFSAATLCLQGTGWTLGERDTESGRRLIVVGGVGSYFRISETQVKGVYNIGWCPIDVCPFCKFDCGIVGGLRENKKIFLALDGNVLPVVFERA

>Glyma.12G234800 (GmKTI28)

MSMRSIGTSLSLMVWLVIATSALAKSDNPPVLDTQGNPLEPGKDYYIKPAITDVGGRVTLLSRNNPCPLYVGQENSDAAEGLPLFFTPFAEEDDVVKVNRDFKVTFSAASICVQGTNWNLAEKDSESGRRLIAASGRDDYFRITETPIKGSYYIGWCPTDVCPFCRFDCGIVGGLRENGKILLALDGNVLPVVFEKAY

>Glyma.15G211500 (GmKTI29)

MSMKLYASLTFVVWLFMATSSLAQYVLDTNRERVDSDDEYYIRPAITDNGGRFTLINRNRSCPLYVGLENTDTPLGYPVKFTPFSRNNNDDDDDDIRVNRDLRVAFDEVSTTCVQSTEWRVGENDTRSGRRLIITGRDETTGSYGNYFRIVETENVGIYNIQWCPTEVCPTCRFICGTGGILRENGRILFALDGTPLPVMFQKKDD

>Glyma.16G211700 (GmKTI30)

MKVSIAKFHSLPLCFFLLLAFNTKPLLAAEPEPVVDKQGNPLEPGVGYYVWPLWADEGGLTLGQTRNKTCPLYVIRDPSFIGTPVSFLAPGLDHVPTLTDLTIDFPVVTVCNQPTVWRLNKVGSGFWFVSTSGDPNDITSKFKIERLEGDHAYEIYSFKFCPSVPGALCAPVGTFEDADGTKVMAVGDDIEPYYVRFQKVSIFGQDKKQPFSIL

>Glyma.16G212100 (GmKTI31)

MKMTLIALLLLVALSTKALLGAAGPAPEQVLDTSGKMVRARTSYYIVPASPDVGGLAMASTGEDCPLDVVAVDGYQGQPLIFTPVNVNKGVIRVSTDLNIYFPIDTSCPLTKAWKLKDYDYSTSQWFVTTGGDFGNPGSQTLANWFKIEKYEDAYKLVYCPSVCKDCSYPCSDIGIYQDQYGKRLALSSEPYRVKFQRAY

>Glyma.16G212200 (GmKTI32)

MKMTLVTLVLVFALITKALAGPASEPVLDALGKKVRADSIYYIVPASSDIGGLASARTDVDCPLDVVAVDGDLGLPLSFTPVNDKKGIIRVSSDLNIYFTSYTIFCPQTTVWKLKYYDDSTSQWFVTTGGELGHPSSQTVANWFKIEKYEDAYKLVYCPSVCSDCNHQCSDIGIYQDQYGKRLALSSEPYKVQFERCWACE

>Glyma.16G212400 (GmKTI33)

MKFMYLAFLLLFVFSSQFLLGGADASPRQVIDTEGKKVRAGVDYYIRPVPTTPCDGRGPCVVGSGYVLIARSSNHTCPLSVAVVEGFRGLAVTFKLVNPKKGVIRVSTDLNIKTSLTNTSCSESTVWKLDAFDDSTGQWFVTTGGVLGNPGKDTIDNWFKIEEYDDDYKLVFCPTVCNFCKPLCRNVGVFRDSNGNQRVALTDEPYKVRFQPSA

>Glyma.16G212500 (GmKTI34)

MKKVSPLAFSILFLAFTIEPFIGIAAAAPEAVLDTSGQKLRTGVKYYILPVFRGKGGGLTVSSSGNNTCPLFVVQEKLEVSKGTPVTFTPYNAKSGVILTSTDLNIKSYGKTTTCDKPPVWKLLKVLTGVWFLSTGGVEGNPGVNTVVNWFKIEKAEKDYVLSFCPSFAQTLCRELGLYVGDDGNKHLSLSDKVPSFKVMFKRA

>Glyma.18G191400 (GmKTI35)

MKSSIPTTTTSTLLTLFLLSAFTSHLPSAGADAVTDRDGAAIRNGGTYHILPLFGVKNGGLELAATRNETCPLTVVQSRTAQIFRGLPVRISSPYRIAYISEGLILNLAFASSPSCAPTPPNWTVVKGLPEGQGVKLLGYGRSTVSGWFKIEKSSLEYLYKLVFCARASKACGEIGISVDDEGINRLVLTEEEGDGIIVEFMKASSVSA

***T. myopaeformis* trypsins**

TmTrp1

>g7808

MLLPTATLSSTAIGIYIFAALLPCYWGQNLTIDKRIIGGKPIQIQRAPFMVNLREDGRFVCGGTLLTRKCVLTAAHCVAGVSTSRLTIQGAVTRLTQSGQMSNVRSVFVPRAYSRRTSNYDVAILRLAKPITNRHARTVKLARRALRPGTRVSVFGWGRSQEDGKFSKSLRAATVRVLPHASCKSRYRNVAVLTRSMFCASMPGVRDSCSGDSGGPVVYRGVQYGIVSWGVGCARRQYPGVNTHIHVVRPWIDSIAAQQC

TmTrp2

>g3594

MLLPTATLSSTAIGIYIFAALLPCYWGQNLTIDKRIIGGKPIQIQRAPFMVNLREDGRFVCGGTLLTRKCVLTAAHCVAGVPTSRLTIQGAVTRLTQSGQMSNVRSVFVPRAYSRRTSNYDVAILRLAKPITNRHARTVKLARRALRPGTRVSVFGWGRSQEDGKFSKSLRAATVRVLPHASCKSRYRNVAVLTRSMFCASMPGVRDSCSGDSGGPVVYRGVQYGIVSWGVGCARRQYPGVNTHIHVVRPWIDSIAAQQC

TmTrp3

>g23695

MALAIANVAARAAGDKVTQTQRVPRLTDNRIVGGVEVDIARAPYQVSLRYKILFDPSYSYSHFCGGSIYSDTVVITAAHCIIGTVPSQFQIAAGTSNCCGGDGIIVPVKDIIMHQDYDPSTAKNDIAIMILAAPLPLNNFTIQAIALTESEVPEGATSIITGWGDTTPSGTPADKLQEVRVPIVSNAACNEDYKNIEVDDSMLCAGLRGVGGKDACQGDSGGPLVVNGKLAGVTSWGYGCALPQYPGVYAKVSHLRPWLLEKLEEYKINLN

TmTrp4

>g23693

MFKIALVGLLLVGIAHGATPTGDLEGRIVGGNDVSIVKHPYQVSVRFKSCNACAYVHECGGSIYNEDTIISAAHCLHHREPRDFVVVAGTDNRVGSDGVVTRIEKIVTHEKYNASITDNDVALLFLATPLPINHVTIERVSLTAETPGTGVKSVVIGWGATTEGGAYSQKLQEAEVAIISKEACQEAYNGGRTITEGMLCARVAGGGKDACKGDSGGPLLVKKELAGIVSWGIGCARPESPGVYADVAYYGEWLRLTITENSLFA

TmTrp5

>g3592

MPFPATDAIRAALVCLLVALASASHKFAALSDTRIVGGVETTISEVPYLVNIRRNGQFSCGGSLITTTCVLTAAHCVRGVTPSSLTIHAGSSRLSQSGQAEQAEKHYVSPFYSSTTLDMDVAIIKLANPLNGPNIATISLCGHSPESEQFVKISGWGITNEYSNAPPDQVRTTSVRVVAKDDCIRAYAGKALLTSTMFCATVAGERDSCSGDSGGPVVYDGRVCGIVSWGFGCARQEYPGVYTNVASRRRHVALAQPRIVGGVTTTIQAAPYLVNLRINGVFFCGGSLVTRQHVVTAAHCVKGKNVGAIVVTAGTSQLDGSGITRSVAKAFLPNKYRRDNENMDVAVLKLQSPINQGNVRPIDLCNTRLKVGNRLTVFGWGVTNENSKTSSNQYRFRARLTRTMFCASVPGSKDSCAGDSGGPAVFGGRLCGIVSFGVGCARRNYAGVYTSCCNRSLILFTLSCA

TmTrp6

>g7809

MSHLIVLVILAVSAIAASATQPPANRARRIVGGDLAVSTQIPYLVNIRKNGVFHCGGSLVTSRCVLTAAHCVRGGAPQDFVVRAGVTFLTDFQNGRRVDQIFTPDQYNNKTLEHDIAVFRLKVAISADRSMKPIRIADFVPKQGELVRVSGWGFTHENATHPPNQMRTVRVRVMQQEQCQALYADYRNVSETMFCASMPGYKDACMADSGGPAVAHGQLVGVVSWGKGHDCGRAESPGVYASLSTMRRIVGGVETTISEVPYLVNIRRNGQFSCGGSLITTTCVLTAAHCVRGVTPSSLTIHAGSSRLSQSGQAEQAEKHYVSPFYSSTTLDMDVAIIKLANPLNGPNIATISLCGHGPESEQFVKISGWGITNEYSNAPPDQVRTTSVRVVAKDDCIRAYAGKALLTSTMFCATVAGERDSCSGDSGGPVVYDGRVCGIVSWGFGCARQEYPGVYTNVASRRRHIALAQPRIVGGVTTTIQAAPYLVNLRINGVFFCGGSLVTRQHVVTAAHCVKGKNVGAIVVTAGTSQLDGSGITRSVAKAFLPNKYRRDNENMDVAVLKLKSPINQRNVRPIDLCNTRLKVGNRLTVFGWGVTNENSKTSSNQYRFRARLTRTMFCASVPGSKDSCAGDSGGPAVFGGRLCGIVSFGVGCARRNYAGVYTSCCNRSLILFTLSCA

TmTrp7

>g23699

MFRLAIVVCALLAAGGARADTHQIGMLPMPDGRIVGGSNADIRQYPHQISMRYKGRHRCGGSIYSANVIVSAAHCVFETDASLITIVAGSTLLSKKNVEIPVLKYIIHSSYKVFNNDYDVAILVLNGRLNFNQYIQPIALARERPATGTEVTVTGWGTLVENGNSPDHLQQVNVNVVDNSNCRKSYLVLLTKRMLCAGVDGGGQDACQGDSGGPLIVNNQLLGIVSWGVGCAQKHFPGVYASVPDLADWIEATVAANTLEFVQT

TmTrp8

>g23186

MTRLAVALLFVSASISAAVPLDAGATAAAAEPEKRIVGGMQTSIAKYPYQVSVRLDSSALIHICGGSIYAPRVIVTAAHCLKGRYASHIRVVAGSSTIADQSEQGVAAKKLIYHSGYSKKTHENDVGLIILAADLVWSDVVQPIPLAPEDPAAGTHATVTGWGKSDEEAKTLTNMLQEVDVKIVDTNVCHAQYTAKDYTITEQMVCAGVEAGGKDTCQGDSGGPMVTSGKLVGIVSWGIGCARSDFPGVYASVPYHADWITAQAQPYL

TmTrp9

>g18287

MTRLAVALLFVSASISAAVPLDAGATAAAAEPEKRIVGGMQTSIAKYPYQVSVRLDSSALIHICGGSIYAPRVIVTAAHCLKGRYASHIRVVAGSSTIADQSEQGVAAKKLIYHSGYSKKTHENDVGLIILAADLVWSDVVQPIPLAPEDPAAGTHATVTGWGKSDEEAKTLTNMLQEVDVKIVDTNVCHAQYTAKDYTITEQMVCAGVEAGGKDTCQGDSGGPMVTSGKLVGIVSWGIGCARSDFPGVYASVPYHADWITAQAQPYL

***H. glycines* trypsins**

*Hg*Trp1

CAA74204

1 MQSLSHILFT FFSILFAAHC QSDESKSAGA QMQTVDENKI RHGKPLTSIV KYPFMATVWQ

61 NDRKLCTASI VSPNYILSAG HCFVKMSEEN YIILVGTVNA KLEKGNGQQF KVEKAHVYSE

121 TVFGQDIAIV KLKNSIDFSD NATQPITLSR RSNFTKTDLA FIAGWGRITD WSSPVTLQGA

181 NVLIWPKDEA RCDGIMESEV CAFGEDGANV CFGDSGGPLL VKSYDGQRWE QIGVTSRGNF

241 VCESNGFFSC VYIYCEWLAK ITEGEVQCLN NE

*Hg*Trp2

CAA74205

1 MRVLLLLCLA LPVFSENIDS HVVGGSNANI ANYPHQLSLR VSGSHSCGAS LVSTTKAVTA

61 AHCGGSAISV YSILGGTTSR TDTTCSTCVT RNLSQFNRHA SFVNNGNQGY PNDVATLHFS

121 AVTTNSNLRT IAMAASSAGD YAGQSCVITG WGRTSATSSL PVTLQQGTMT VLTNSNCASR

181 WSAAQINNGH ICVSSSSVSA CSGDSGGPLV CGSTLVGATS WGQAQCNPSY PSVYTRISYF

241 RSWIDSH

***Bactrocera oleae* Trp (*Bo*Trp1)**

XP_014094233

MYSVSTNNAMAFKLKLIFQLVIYLSWHHMLCTGIGERIVGGSSVTEKKYTYFVRVHYEGILWCGGSLVRNNAVVTAAHCVSDVNVKELRVHADTISLRDEGIVRQVKKVVISSLYNERTTNYDVAVLILASAIPNSSFTPIQLQKTPVAAGTKCLVIGHGNTKENGKIPTQLQEVWVPVLSRNVCQRRYAGVARITRSMLCAWESGKDSCGGDSGGPMVCNGQQAGIVSWGVECADSRYPGVYTDISSVYTFIVRTLQRYQ
